# Thymoquinone as a natural spermostatic substance in reproductive medicine: An experimental study

**Published:** 2017-10

**Authors:** Farhad Golshan Iranpour, Khatereh Fazelian, Gholam Reza Dashti

**Affiliations:** *Department of Anatomical Sciences, Isfahan University of Medical Sciences, Isfahan, Iran.*

**Keywords:** Nigella sativa, Sperm immobilizing agents, Sperm motility, Thymoquinone

## Abstract

**Background:**

Nonoxynol-9 a nonionic surfactant is widely used for its spermicidal effects. Finding new sperm immobilizing agents is necessary because Nonoxynol-9 damages the tissues of female reproductive system.

**Objective:**

The aim of this study was to evaluate the effects of Thymoquinone (TQ) as a potential spermostatic compound on the motility and viability of human spermatozoa.

**Materials and Methods:**

In this experimental study, the effects of 5, 10, 20, 50, 100 µg/ml, 1 and 10 mg/ml of TQ on normozoospermic semen samples were investigated. Sperm motility and viability were compared between untreated and TQ-treated aliquots of each semen sample. To evaluate the effects of TQ on the alteration of mitochondrial membrane potential (MMP), 32 semen samples were examined using 50 µg/ml of TQ. Flow cytometric analysis was performed after staining of spermatozoa with JC-1.

**Results:**

Doses above 20 µg/ml of TQ could eventually immobilize all spermatozoa in culture medium. Adding 50 µg/ml of TQ did not significantly diminish the percentage of viable spermatozoa and flow cytometry results revealed that this amount of TQ could decrease sperm MMP.

**Conclusion:**

TQ could discontinue the movement of sperm cells in medium without reducing the population of live spermatozoa. It is more likely that TQ exerts its spermostatic action by mitigating the MMP of spermatozoa. Therefore, TQ could be considered as a potential new natural spermostatic chemical.

## Introduction

With the high cost and the probable side effects of synthetic drugs, scientists and researchers are trying to substitute them with herbal drugs and natural components. Finding cheap, easy to use and effective spermicides is necessary to avoid unwanted pregnancies. These chemicals should also be able to prevent the transmission of sexually transmitted diseases. One of the candidates for such a product is nonoxynol-9 (N9). This surfactant is an approved spermicide which has been widely used for around 50 yr in a variety of contraceptive barriers and creams. Some reports indicate that N9 increases the frequency of genital lesions and offers no protection against HIV. Furthermore, N9 kills spermatozoa by disrupting the integrity of their plasma membrane and the non-specific nature of this mechanism of action injures the tissues of the lower female reproductive tract and as a result, women become more vulnerable to sexually transmitted diseases (1, 2).

**Figure 1:**
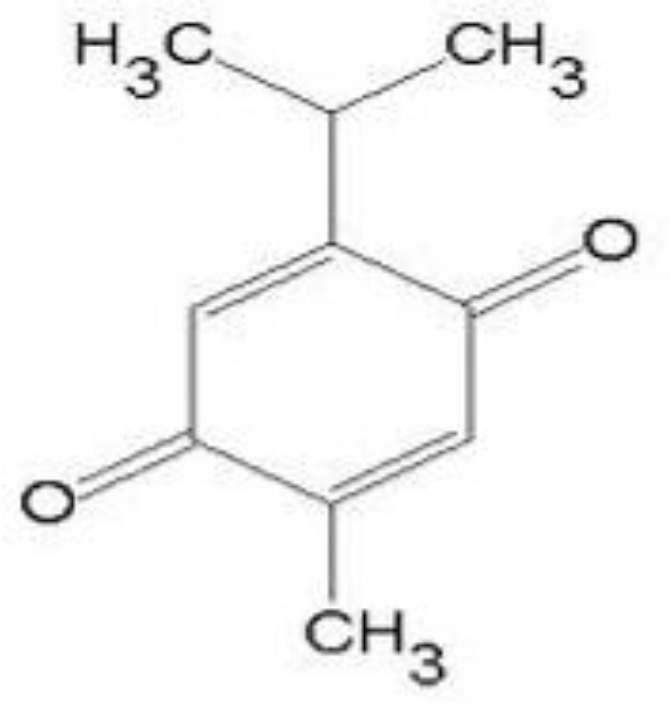
Chemical Structure of Thymoquinone.

Nigella sativa (black seed) is one of the annual herbaceous plants belonging to the Ranuculacea family. Thymoquinone (2-isopropyl-5-methyl-1, 4-benzoquinone) (TQ) is the main constituent of the volatile oil from Nigella sativa seeds (Figure 1). It has been reported that TQ is an anticancer, anti-diabetic, anti-oxidant and anti-inflammatory agent (3, 4). 

TQ protects mouse and rat testis against the hazardous effects of heat stress, cyclophosphamide and toluene (4-6). Also, Nigella sativa oil led to an improvement in histology and function of seminal vesicles and prostate gland in nicotine treated rats (7). On the basis of these results, it would be expected that TQ will exert beneficial effects on sperm, such as increasing its motility in-vitro. Hughes and colleagues suggested that quinones are compounds that can induce a state of “spermostasis” characterized by the extremely rapid inhibition of sperm movement without compromising cell viability (8). Since TQ belongs to the quinones' family, it may be a potentially spermostatic compound that exclusively affects sperm. Alhimaidi reported that TQ immobilizes mouse spermatozoa and hereby inhibits the fertilization of oocytes and embryo development after IVF and ICSI (9). In contrast, Kamarzaman *et al* demonstrated that adding TQ to culture medium improves fertilization rate and reduces defected blastomeres and fragmented embryos following mouse paternal and maternal exposure to cyclophosphamide (10). The aforementioned results reflect the substantial inconsistency of TQ effects on spermatozoa in the culture medium. 

There is an urgent need to find safe, natural and dual action contraceptives that combine the prevention of pregnancy with protection against sexually transmitted diseases. Due to the necessity of finding new contraceptives that exclusively affect sperm and the lack of comprehensive study about the neat effects of TQ on human sperm cells.

The aim of the present study was to examine the effects of TQ as a potential spermostatic compound on the motility and viability of spermatozoa in the culture medium.

## Materials and methods

**Chemicals**

All chemicals were supplied by Sigma-Aldrich (St.Loius, MO, USA) unless stated otherwise.

**Experimental design**

In this experimental study, semen samples were obtained from 70 normozoospermic men attending Andrology Unit of Shahid Beheshti Hospital, Isfahan, Iran. Semen samples were classified as normozoospermic according to WHO criteria (11). 5, 10, 20, 50, 100 µg/ml, 1 and 10 mg/ml doses of TQ were applied. Ten semen samples were randomly chosen for each dose of TQ. 

**Semen collection and preparation**

Semen specimens were collected by masturbation in sterile containers after 3-7 days of sexual abstinence. All semen samples were allowed to liquify for 30 min at 37^o^C. Within 1 hr of collection, routine semen analysis was performed using computer-assisted sperm analysis system (CASA, VT-Sperm Test, 2.3 model-Company of Video Test-Finland) according to the World Health Organization guidelines (11). The semen samples were washed twice and resuspended in Modified Ham’s F10 with %5 human serum albumin (Irvine Scientific, Santa Ana, California). One 0.5 ml aliquot of each semen sample was considered as treated sample by adding the desired dose of TQ while another 0.5 ml aliquot of the sample was considered as an untreated sample without adding TQ. Then, both the samples were incubated at 37^o^C. After incubation for 1 hr, the percentages of total motility and viability of spermatozoa were assessed in both untreated and treated samples.

**Preparation of TQ solutions**

TQ was dissolved by an initial addition of dimethyl sulfoxide (DMSO), followed by distilled water and stored at 4^o^C. The final concentration of DMSO was <0.5% that shows no significant toxic effects in-vitro (12). Further dilution was done in Modified Ham’s F10 supplemented by 5% Human Serum Albumin. 

**Sperm motility**

The percentage of total motile spermatozoa was evaluated in untreated and treated samples by a light microscope equipped with CASA system (CASA, VT-Sperm Test, 2.3 model-Company of Video Test-Finland). Moreover, the proportions of fast progressive, slow progressive, non-progressive and immotile spermatozoa were measured in untreated and treated samples of 5 and 10 µg/ml of TQ. Fast progressive sperm cells are those that swim forward in a straight line. Slow progressive spermatozoa swim forward, but either in linear or curved line. Non-progressive sperm cells move their tails but do not move forward and immotile spermatozoa do not move at all. Then, the results of sperm motility were compared between untreated and treated samples.

**Sperm viability**

Viability was evaluated by Eosin staining method (11). Three slide replicates were assessed by an expert laboratory technician. 0.05% Eosin Y solution was prepared in 0.9% NaCl (0.9 gram of NaCl to 100 ml purified water). Briefly, 5 µl of sperm sample was mixed with 5 µl Eosin Y solution on a microscopic slide. After putting cover slip, the slide was examined by light microscope under ×400 magnification. At least 200 sperm cells were counted and the proportion of unstained (alive) cells was calculated. The results were compared between untreated and treated samples. 

**Flow cytometric assessment of mitochondrial membrane potential (MMP)**

To examine MMP changes of sperm after using 50 µg/ml of TQ, 32 semen samples were studied. Two 0.5 ml aliquots of sample were prepared as described before. One of the aliquots was contained medium alone as an untreated-50 sample and the other one was contained 50 µg/ml of TQ as treated-50 sample.

Sperm MMP was evaluated by using the cationic lipophilic stain5, 5', 6, 6’-tetrachloro 1, 1’, 3, 3’-tetraethylbenzimidazolyl carbocyanine iodide (JC-1). The JC-1 staining kit was purchased from Sigma-Aldrich and was used according to the manufacturer’s instructions. Briefly, a stock solution of 1 mg/ml JC-1 was prepared in dimethyl sulfoxide and stored at -20o C until use. Untreated-50 and treated-50 aliquots were diluted to a sperm concentration of 1-2 million per aliquot with a working solution of JC-1 in phosphate-buffered saline (PBS; final concentration 1 µg/ml) and incubated in dark for 15 min at 37^o^C. After incubation, sperm cells were analyzed with a FACS Calibur flow cytometry (Becton Dickinson, Heidelberg, Germany). The JC-1 monomeric form accumulates in low MMP mitochondria, emitting green fluorescence and is detected through the FL1 channel (530±30 nm band-pass filter).

In contrast, JC1 forms aggregates in high MMP mitochondria and they are detected through FL2 channel (582±42 nm). Two controls were used to evaluate the probe’s response to changes in mitochondrial status: 1) sperm cells were identically processed for each fraction except that stain was replaced with 10 µl PBS; 2) sperm cells were processed in the presence of 1 µM valinomycin for 60 min at 37^o^C. Vanilomycin permeabilizes the mitochondrial membrane for potassium ions, and thus, dissipates the mitochondrial electrochemical potential and maybe used as a control that prevents JC-1 aggregation.

**Ethical consideration**

Ethical committee of Isfahan University of Medical Sciences approved all procedures performed in this study involving human participants (etichal code no. 392393). Informed consent was obtained from all individual participants included in the study.

**Statistical analysis**

The statistical analysis was carried out using SPSS software for windows (Statistical Package for the Social Sciences, version 19, SPSS Inc., Chicago, Illinois, USA). The data are expressed as mean±SD (standard deviation). All variables were checked for normal distribution and one-way analysis of variance (ANOVA) followed by a Duncan test or paired t-test was used to compare the results, as appropriate. A p-value<0.05 was considered statistically significant.

## Results

**The effects of thymoquinone on sperm motility**

Statistical analysis illustrated that there were not significant differences between the mean percentages of total sperm motility in untreated samples of all doses of TQ. The mean percentages of total motility were significantly increased in treated 5 and 10 µg/ml samples (p=0.037 vs. p=0.02) in comparison with untreated samples, whereas doses above 20 µg/ml cause the total immobilization of spermatozoa in culture medium (p<0.001). The results are summarized in table I.

**The effects of lower doses of Thymoquinone on sperm motility**

**Figure 2:**
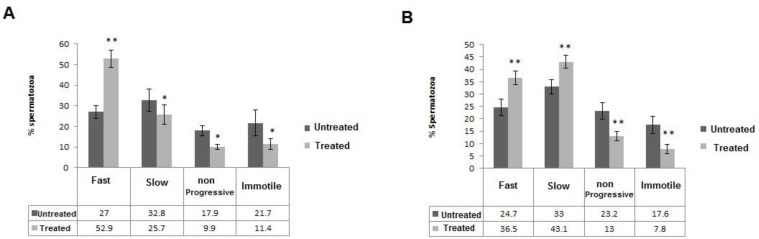
Different categories of motile and immotile sperm cells in untreated and treated aliquots of 5µg/ml (A) and 10 µg/ml (B) of TQ. Each bar is the mean (±SD) of percentages of fast progressive, slow progressive, non-progressive and immotile spermatozoa.

The percentage of total motility of spermatozoa was significantly increased after addition of 5 and 10 µg/ml of TQ (Table I). The main results related to the effects of 5, 10 µg/ml doses of TQ on the proportion of fast progressive, slow progressive, non-progressive and immotile spermatozoa are summarized in Figure 2. The percentages of fast progressive sperm cells were augmented significantly in treated 5 and 10 µg/ml samples in comparison with untreated ones and this increase was higher in the 5 µg/ml vs.10 µg/ml treated samples (52.9% p<0.001 vs. 36.5%, p<0.001, respectively). In both doses of TQ, the percentage of fast progressive spermatozoa increased along with decreasing the percentages of non-progressive (9.9%, p=0.02 for 5 µg/ml and 13%, p<0.001 for 10 µg/ml) and immotile spermatozoa (11.4%, p=0.01 for 5 µg/ml and 7.8%, p<0.001 for 10 µg/ml). However, the mean percentage of slow progressive motile spermatozoa was reduced in 5 µg/ml treated samples (25.7%, p=0.01) and augmented in 10 µg/ml treated samples (43.1%, p<0.001).

**The effects of Thymoquinone on mitochondrial membrane potential**

We found that 50 µg/ml of TQ is a dose that could immobilize all spermatozoa without making significant changes in the percentage of viable sperm cells. Therefore, MMP alteration in this dose is not related to apoptosis changes of sperm and might be related to the immobilizing effect of TQ on spermatozoa. 

**Figure 3:**
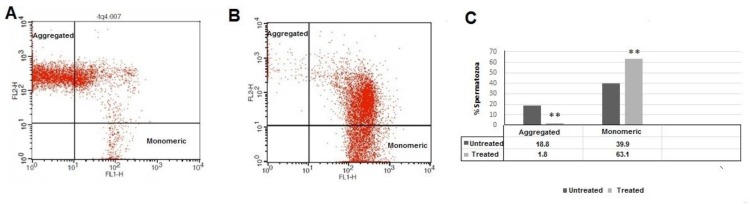
Cytograms of FL1 (green fluorescence) and FL2 (red-orange fluorescence) of spermatozoa from an untreated-50sample (A) and a treated-50 sample (B).Mean percentages of aggregated and monomeric spermatozoa in untreated-50 and treated-50 samples.

Representative flow cytometric results from untreated-50 and treated-50 samples are shown in Figure 3. The proportion of sperm cells with aggregated JC-1 (higher MMP) was significantly higher in untreated-50 than treated-50 samples (18.8±20.02 vs. 1.8±4.4, p<0.001, Figure 3). In contrast, the proportion of sperm cells with monomeric JC1 (lower MMP) was significantly lower in untreated-50 than treated-50 aliquots (39.9±22.05 vs. 63.1±19.4, p<0.001, Figure 3).

**The effects of higher doses of Thymoquinone on sperm motility**

According to our data, doses above 20 µg/ml could stop spermatozoa in culture medium after a while. In 20, 50, 100 µg/ml and 1, 10 mg/ml treated samples the mean percentages of motile spermatozoa gradually decreased to zero. The differences between the total motility percentages of treated and untreated samples in all doses of TQ were significant (p<0.001). The higher doses of TQ could stop spermatozoa in a shorter period of time. This time was 50, 35, 25, 2 and lower than 2 min for 20, 50, 100 µg/ml and 1, 10 mg/ml of TQ respectively. 

**The effects of Thymoquinone on sperm viability**

Similar to motility, the mean percentages of sperm viability were not significantly different among untreated samples of all doses of TQ. The mean percentages of viability were significantly decreased in 20 µg/ml, 100 µg/ml, 1 mg/ml and 10 mg/ml of TQ in comparison with untreated samples (p<0.001 for all of these doses). There was no significant difference in the percentages of viability between untreated and treated samples in 5 µg/ml (p=0.49), 10 µg/ml (p=0.1) and 50 µg/ml (p=0.21) of TQ. The lowest decrease in viability was seen in 5, 10 and 50 µg/ml treated samples. However, the decrease of viability in 100 µg/ml, 1mg/ml, and 10 mg/ml treated samples (doses higher than 50 µg/ml of TQ) was 20, 11.6 and 11.1% respectively in comparison to untreated samples (Table I).

**Table I:** Total motility and viability of spermatozoa in untreated (control) and treated (case) samples.

**Dosage**	**Motility**		**Viability**	
	**Untreated**	**Treated**	**Untreated**	**Treated**
5 µg/ml	77.6 ± 5.6	88.5 ± 2.01 *	58.7 ± 2.7	57.1 ± 2.5^NS^
10 µg/ml	80.9 ± 4.6	92.6 ± 2.4*	60.8 ± 2.4	58.2 ± 2.9 ^NS^
20 µg /ml	67.2 ± 8.4	00.0 ± 0.0**	65.2 ± 9.7	51 ± 10.02**
50 µg/ml	72.2 ± 10.8	00.0 ± 0.0**	66.1 ± 6.5	62.7 ± 5.07 ^NS^
100 µg/ml	70.1 ± 9.7	00.0 ± 0.0**	64.8 ± 11.02	48.8 ± 11.9**
1 mg /ml	67.7 ± 7.9	00.0 ± 0.0**	65.9 ± 5.8	54.3 ± 6.7**
10 mg/ml	73.8 ± 10.7	00.0 ± 0.0**	62.7 ± 5.3	51.6 ± 3.2**

Data are presented as mean±SD.

NS=Not Significant from untreated group

* Different from untreated group, p<0.05

** Different from untreated group, p<0.001

* Different from untreated group, p<0.05; **Different from untreated group, p<0.001.

**Different from the untreated group, p<0.00 (C).

## Discussion

In the present study, our findings demonstrated that TQ is able to immobilize sperm cells but this effect is dose-dependent. In fact, doses over 20 µg/ml of TQ have the potential to cease sperm motility in the culture medium. Similar results have been reported by Hossain *et al* (13). They found that the low concentrations of TQ stimulated the growth of cancer cells whereas high concentrations of TQ ranging from 50-100 µg/ml, inhibited the growth of these cells in culture medium. The only explanation for this phenomenon is that TQ in low concentrations does not directly act on cells and exerts its effects by its antioxidant activity. Some reports indicate that reactive oxygen species (ROS) induce sperm motility reduction. The reduction in motility may have been due to a ROS- induced lesion and it seems that lower doses of TQ improve sperm motility by its antioxidant effects while higher doses of TQ are effective doses that directly affect sperm cells and their motility by its spermostatic properties (14). Finding the exact cause of this phenomenon needs further investigation.

The results obtained from previous in-vitro experiments on TQ have a huge difference with each other. Kamarzaman and colleagues used 1, 10, and 100 μM (0.165 µg/ml, 1.65 µg/ml, and 16.5 µg/ml) of TQ in fertilization medium of mouse oocytes following paternal and maternal exposure to cyclophosphamide and TQ improved the fertilization rate of the oocytes (10). Unlike Kamarzaman *et al* Alhimaidi reported that the applying of 10 mg/ml of TQ in sperm suspension and embryo treatment medium immobilized all mouse spermatozoa and also reduced fertilization rate and embryo development in-vitro (9). The results of the present study can explain the differences between the obtained results of Kamarzaman and co-workers and Alhimaidi. 

Our results are in agreement with Kamarzaman *et al* because their selected doses are under 20 µg/ml. According to the present study 5 and 10 µg/ml of TQ increased the percentage of motile sperm cells. Thus, lower doses of TQ may improve the results of in vitro fertilization because our data demonstrated that these doses augment the percentages of fast progressive motile spermatozoa in the culture medium. Furthermore, doses above 20 µg/ml of TQ, such as 10 mg/ml that was used by Alhimaidi, immobilized spermatozoa in the culture medium; therefore, may lower the rate of in vitro fertilization.

The evaluation of sperm viability in untreated and treated samples showed that 50 μg/ml of TQ immobilizes sperm cells but does not significantly reduce the percentage of viable spermatozoa. In a similar way, higher doses of TQ stop sperm cells and although these doses decrease the percentage of viable spermatozoa but even for 1 mg/ml and 10 mg/ml doses, reduction in live sperm population is about 11%. However, among different doses of TQ that were examined in our experiment, 50 μg/ml is a dose that all sperm cells were immobilized in it but the significant differences in sperm viability were not seen between untreated and treated samples. 

The percentage of viable sperm cells did not show any significant differences in untreated-50 and treated-50 aliquots and this fact indicates that the alterations of MMP in this dose should not be related to apoptotic effects of TQ. Therefore, we chose this dose for the investigation of MMP alteration after TQ application and its relation to sperm motility. Previous works have found that normal cells such as pancreatic ductal epithelial cells and mouse keratinocytes show resistance to the apoptotic effects of TQ (13, 15-17). Furthermore, Hughes and colleagues suggested quinones as a class of in-vitro spermostatic agents that act specifically on sperm and have anti-microbial activity with a minimum of cytotoxicity as well (8). Spermostasis is a state that is characterized by rapid inhibition of sperm movement without killing it. As TQ belongs to quinones, it may act as a spermostatic which is in agreement with the results of the current study.

TQ is an abundant component of Nigella sativa oil extract and our results indicate that it can act as a potential spermostatic agent. Currently, N9 is a spermicidal detergent that is used in a wide variety of contraceptive barriers and creams. At first, N9 was suggested as the inhibitor of HIV virus in-vitro (18), but later it was shown that N9 facilitates transmission of HIV by the irritation of vaginal mucosa (1, 2). It disrupts sperm plasma membrane and nonspecifically kills it, so it also can damage the tissues of the lower female reproductive system. Satisfying contraceptive should specifically act on sperm and at the same time has no effect on the mucosal cells of the vagina. Another feature that should be expected from a rewarding contraceptive is the inhibition of sexually transmitted diseases. 

Azeiz *et al* examined the in vitro effects of TQ on vaginal candidiasis in prednisolone-induced immune suppressed mice. Their results showed the effectiveness of TQ against vaginal candidiasis. Moreover, TQ showed no effect on vaginal cells (19). Several studies indicate that TQ has no significant effect on normal cells in-vivo (20-22). Also, Nigella Sativa extract seems that have the low level of toxicity in-vivo (23). The antimicrobial, the antifungal and the antiviral effects of Nigella Sativa and TQ have been revealed by many studies (23, 24). Hughes *et al* suggested that quinones have powerful inhibitory effect against Chlamydia muridarum. The proposed mechanism of action was inactivation of major outer membrane proteins by quinines (8). This mechanism is quite similar to that is mentioned by Azeiz and co-workers (19). Another study revealed the antitrichomonal effect of Nigella sativa extract (25). Therefore, TQ shows spermostatic activity as well as antibacterial and antifungal effects. Furthermore, TQ is non-toxic for normal and vaginal cells, so it may not irritate vaginal epithelium.

Ruiz-Pesini *et al* showed the close relationship between sperm motility and mitochondrial enzyme-specific activities (26). Also, several previous reports acknowledged the importance of MMP in sperm motility and showed that the changes of MMP occur in parallel or before changes in sperm motility (27-29). Our flow cytometric results revealed that TQ can change inner mitochondrial membrane potential and this effect may play a major role in sperm immobilization. On the basis of our results, 50 μg/ml of TQ is a dose that immobilizes all sperm cells but does not kill them. By the application of this dosage, the inner membrane potential of mitochondria decreased and the proportion of spermatozoa with monomeric JC1 increased. Some reports emphasize that impaired mitochondrial action demonstrated by a reduction in MMP is related to reduced sperm motility and its reproductive ability (29-32). Hughes *et al* declared that quinones target A Kinase-Anchoring Proteins (AKAP3 and AKAP4) function and as a consequence, suppress the abilities of cAMP to drives protein kinase A-dependent activities in sperm tail and in this way quinones exert their spermostatic action on sperm (8). However, the exact mechanism of TQ action on sperm motility needs further investigation.

## Conclusion

TQ has a dose dependent effect on sperm motility which higher doses are spermostatic. To the best of our knowledge, ours is the first study describing TQ as a novel natural spermostatic agent.
